# Social Network, Sense of Responsibility, and Resident Participation in China’s Rural Environmental Governance

**DOI:** 10.3390/ijerph19116371

**Published:** 2022-05-24

**Authors:** Haibo Ruan, Jun Chen, Chao Wang, Wendong Xu, Jiayi Tang

**Affiliations:** 1 Institute of China Rural Studies, Central China Normal University, Wuhan 430079, China; ruanhb@mails.ccnu.edu.cn (H.R.); chenjunccnu@163.com (J.C.); 2 School of Public Policy & Management (School of Emergency Management), China University of Mining and Technology, Xuzhou 221116, China; wangchaoccnu@163.com; 3 School of Foreign Studies, China University of Mining and Technology, Xuzhou 221116, China; xwdspace@163.com; 4 School of International Relations & Public Affairs, Fudan University, Shanghai 200433, China

**Keywords:** social network, sense of responsibility, rural residents, rural environmental governance

## Abstract

Based on a survey of 2343 rural residents in China, this paper adopts a binary logistic regression model as the analytical tool to study the impact of rural residents’ social network and sense of responsibility regarding their participation in environmental governance. The results show that the cost, frequency and scope of social network activities have positive and significant influences on resident participation in rural environmental governance. The cost of a social network is conducive to building a rural social network, enhancing the connection of interests and promoting the formation of a rural community. Extending social network objectives from family members to villagers can improve the cultural identity and emotional identity of rural residents. The increase in the frequency of social network activities can not only enhance trust among residents, but also reduce the cost of environmental governance mobilization. The scope of a social network acts as an inhibitor whereby social interaction beyond the scope of rural areas will reduce identification with rural emotions. The four dimensions, including responsibility cognition, responsibility will, responsibility emotion and responsibility behavior have significant influences on resident participation in rural environmental governance. Residents’ sense of responsibility plays the role of an introverted driving force for them to take part in rural environmental governance, which itself helps to overcome “non-participation” behaviors of “rational smallholders” to a certain extent. Furthermore, it endows rural environmental governance with resilience. So, it is of significance to enhance rural residents’ social networks and to improve rural residents’ cognition of collective responsibility.

## 1. Introduction

In 2015, the United Nations called for Sustainable Development Goals (SDGs) as a global program planned to ensure that both rural and urban populations enjoy health by 2030. The concerted efforts of countries around the world have contributed to the development of global health which is manifested in increased life expectancy at birth and healthy life expectancy, along with reduced child mortality. However, human beings still face various environmental health problems. The World Health Statistics 2021 report released by the World Health Organization (WHO) shows that rural and remote areas have poorer environmental governance than cities. In addition, there is a great imbalance in environmental governance between urban and rural areas, which is mainly manifested in the unequal distribution of health resources. In China, there is also an imbalance between rural environmental governance and economic development. As shown in Table 1, the per capita disposable income of rural residents in 2020 reached 17,131 RMB, an increase of 4768 RMB compared to 2016. The per capita disposable wage income of rural residents increased from 5022 RMB in 2016 to 6974 RMB in 2020. The gross output value of agriculture, forestry, animal husbandry and fishery increased from 10.0647873 billion RMB in 2016 to 13.778217 billion RMB in 2020. At the same time, the gross output value of agricultural, forestry, animal husbandry and fishery was on the rise.

The rapid development of the rural economy and agricultural production causes pollution in livestock and poultry breeding, as well as polluted water and farmland. According to the information released in China’s Second National Survey of Pollution Sources bulletin, the amount of pollutants discharged from agricultural source water in 2017 was 10,671,300 tons of chemical oxygen demand, 216,200 tons of ammonia nitrogen, 1,414,900 tons of total nitrogen and 21,200 tons of total phosphorus. The amount of plastic film used reached 1,419,300 tons, and the accumulated residue reached 1,184,800 tons. These actually reflect the imbalance between environmental governance and economic development. Meanwhile, rural development in the economy has also brought about changes and transformations in the structure of rural social networks. On the one hand, in terms of geographical scope, the methods and objectives of residents’ social network activities have expanded significantly. The development of internet communication technology has changed traditional face-to-face communication in rural areas. It breaks through the limitations of time and space, makes foreign exchanges possible, improves the frequency and efficiency of exchanges and reduces the cost of exchanges [1]. On the other hand, with the changing nature of social interaction and the developing market economy, more rational choice factors have been incorporated into the rural residents’ social interaction. The cultural, emotional and value factors behind the interaction have also transformed [2]. The development of the economy in rural areas not only brings environmental governance problems to the countryside, but also brings about changes in residents’ social networks. Taylor’s research shows that residents’ social networks—such as partnerships—influence their environmental governance behavior [3]. So, can a social network influence the participation of rural residents in rural environmental governance?

Different from conventional ruling and management, governance refers to the coordination and cooperation of multiple subjects on the basis of common goals to jointly handle public affairs [4]. In February 2018, China’s central government issued the “Three-Year Action Plan for the Improvement of Rural Human Settlements”, which proposed to carry out centralized improvement of rural garbage, sewage treatment and village appearance. Rural environmental governance refers to the rural organization of residents to participate in rural household waste and domestic sewage treatment, toilet manure treatment, village appearance management, etc., aiming to improve the living conditions of rural residents and improve people’s health. The participation of rural residents in rural environmental governance means that residents participate in the governance of the rural public health environment under the organization of rural managers [5]. Rural environmental governance, with its nature of “public goods”, has a positive external effect on residents who can enjoy the results of governance without paying any cost. However, rural environmental governance faces a series of problems, such as a lack of governance entities, backward environmental governance technology, a shortage of resource elements, and high governance costs. Therefore, in order to avoid the phenomenon of a “tragedy of the commons”, it is necessary for residents to play the main role and participate in rural environmental governance. At present, the traditional way of operation for social network activities in rural areas is being transformed, from the communication between acquaintances to the communication between strangers, from human interaction to market transactions, homogeneous communication and heterogeneous communication [6]. Since the objectives, scope and methods of interaction in social networks among rural residents have all changed significantly, how does the changing of social networks in rural areas affect residents’ participation in rural environmental governance?

Research on the influencing factors of rural residents’ participation in rural environmental governance focuses on the following aspects. The first focus is on resident individual and family factors. Studies have found that factors such as the education background, gender and age [7] of residents significantly affect their participation in environmental governance. Wang et al. found that rural residents with better economic conditions have a higher willingness to participate [8], but Min et al. believe that there is not a complete positive correlation between household economic conditions and improved behavior, and that those with strong economic backgrounds are more likely to take part in “non-participation” behavior [9]. The second focus is about the environmental protection awareness of rural residents. Based on the theory of planned behavior, Wang et al. found that the concept, attitude, enthusiasm and self-efficacy of rural residents toward environmental protection affect their environmental protection behavior [10]. However, Qing et al. believe that sensitive environmental pollution tolerance does not promote environmental improvement behavior [11]. Zhang et al. studied the influence of social capital on villagers’ participation activities in rural environmental governance, and their research results showed that rural social norms, social trust and social networks all had a significant impact on residents’ participation in governance behavior [12]. Reed et al. believe that in areas without a specific culture, residents lack a sense of cultural identity, resulting in a lower probability of their participation in environmental governance [13]. The fourth focus is the environmental governance training factor. The study by Boudaghpour [14] found that training rural residents in environmental hygiene knowledge can increase their motivation to participate. Carrard believes that training rural residents on the concept of circular development can encourage them to adopt environmentally friendly behaviors [15].

Scholars have analyzed the factors influencing residents’ participation in rural environmental governance from different perspectives (such as cognitive behavior and planned behavior), which is significant to this study. However, rural resident participation behavior itself is the result of comprehensive influence. Meanwhile, the above research did not take into account the characteristics of China’s rural areas; that is, the acquaintance society. The behavior logic of villagers is guided by their local behavioral logic. The understanding of rural residents’ behavior cannot be simply taken from the “individual-based” perspective, but from the “relation-based” perspective [16]. In the acquaintance society, the relationship formed by rural residents’ communication affects their behavior, and communication is the basis of the relationship. Rural residents change their behavior based on acquiring information, constructing social capital and obtaining social resources in a social network.

In 1966, Boulding proposed the concept of circular economy [17]. He compared the development of the human economy to a large spaceship which consumes natural resources and emits waste in the process of production and consumption, and the toxic substances discharged from the waste will destroy the living space and lead to the collapse of the social system. Therefore, the development of a human economy needs to follow the idea of an ecological economy. In 1997, Frosch developed the theory of circular economy. He believed that the development of industry needs to reduce or eliminate the impact of industrial activities on the environment and establish a harmonious ecosystem [18]. Therefore, the development of circular economy requires enterprises to undertake environmental protection responsibilities, and enterprises should follow the social environmental protection responsibilities of ecological design, reuse, low energy consumption and zero emissions in the production process [19]. For residents living in the countryside, protecting the rural environment also requires them to fulfill their environmental protection responsibilities. Based on this, this paper intends to study the influence of a social network and sense of responsibility on rural residents participating in behaviors related to environmental governance. In theory, it is expected to contribute to the current theories of social networks, especially the impact of different aspects of social networks on resident participation in environmental governance. From the perspective of policy, the author puts forward suggestions for improving rural residents’ participation in environmental governance.

## 2. Theoretical Background and Hypothesis Development

The government-launched living environment improvement plan includes many aspects, such as toilet reform, sewage treatment, rural garbage treatment and village outlook improvement. This paper mainly focuses on the behavior of rural environmental governance, specifically the governance of rural domestic waste. These household wastes include solid waste, paper waste, plastic waste, metal waste and other wastes generated during daily life in rural areas [20]. Therefore, rural environmental governance, in this research, refers to the governance initiated by the government to deal with public waste in rural areas [21]. For individuals, family environment governance belongs to the private domain in which they positively participate to maintain. However, environment management behavior beyond the scope of family has a nature of public good. Rational people will choose to avoid rather than participate in governance. It is necessary to take into consideration the impact of social networks and a sense of responsibility on individual behavior. In relational society, rural residents’ communication is the “cultural network of power” formed by relatives, friends, clans, beliefs, etc., which is amid a variety of formal and informal communication. The interactions of rural residents produce different communication types which are beneficial to the generation of rural residents’ positive behavior [22]. Therefore, it is necessary to explain the influence of social networks on rural resident participation behavior.

### 2.1. Social Networks

Social network theory comes from sociology. Social networks were formally proposed by Barnes in 1954 to represent relationships in society [23]. Mitchell defines the concept of a social network, believing that it is the connection between a group of specific actors, including direct connections between individuals and indirect connections formed by individuals communicating and sharing material resources through the external environment. These connections, which can be divided into formal and informal connections, are used to explain the social behavior of individuals [24]. A social network is a relational system and relational structure. In the social network structure, each actor has more or less of a relationship with other actors. In 2002, Isham and Kahkonen proposed measuring the popularity of social networks and the “neighbor trust index” [25]. In 2004, Peng discussed the influence of informal communication on rural development based on social network theory [26]. Due to differences in the understanding of social networks by different scholars, social network theory has formed three research directions in the development process.

The first direction is on the weak ties and embedding theory represented by Granovetter. Granovetter divides the relationship between network members into strong ties and weak ties according to the depth of affection, intimacy, the length of communication time and whether there is reciprocal behavior. Strong ties are relatively close social relationships formed by frequent interactions between individuals, while weak ties are indirect relationships between network members and other individuals [27]. The second is the structural hole theory represented by Burt. The structure of a social network is divided into “structure with holes” and “structure without holes”; “structure with holes” means that the individual loses contact with other members, while “structure without holes” means that the individual maintains a close relationship with other members. “Structure without holes” can help individuals obtain useful information, resources, etc. from social networks [28]. The third is the social capital theory represented by Lin and Putnam. Lin emphasized that individuals acquire social resources such as wealth, power and prestige in social networks through social relationships [29]. Putnam believes that social capital includes social networks, norms and trust, and a social network is one of the elements that constitutes social capital [30].

Although scholars of different kinds pay attention to different perspectives, scholars of social network theory all believe that the actor’s position in the social network and the social relationship formed have a certain impact on the actor’s behavior [31]. Chinese social network research originates from the theory of “Pattern of Difference Sequence” proposed by Fei. China’s social network relies on the inherent blood and geographical relations of the rural society. The village has a relationship with scarce social resources such as rights, status, property and marriage. The behavior of individual actors is affected by the relationship between intimacy, thickness, distance and so on in the social network [32]. Through interpersonal contact and interaction, it helps individuals to establish social networks, which is conducive to the accumulation of their own social capital. Through social network connections, individuals can improve their social participation behavior [33]. Social relationships guide individuals’ behavioral direction. After repeated reinforcement, it becomes an internalized individual code of conduct. In terms of the division of different dimensions of social interaction, this paper draws from Chen’s method to divide social networks into four classifications: cost, objective, frequency and scope [34].

Social network cost refers to the time, capital, trust and other costs that individuals pay in the process of communication in order to integrate or build a specific social structure which constitutes the investment of social communication [35]. In a society of acquaintances, the most typical social interactions are ritual human interactions, such as marriage, birthday celebrations, and festival celebrations, which consume much time and money. These personal exchanges need to pay the price of time and gift money. Ritual human interactions are endowed with symbolic meanings. The cost of human interaction has become one of the criteria for measuring the intimacy of the relationship between the two. The scale of the ritual in turn becomes an indicator of the scale and scope of the family social network [36]. The richer the social relations of villagers, the more investment in social communication. This model is helpful for villagers to establish their identity within rural culture and customs. By accepting others emotionally and forming emotional connections rather than purely interest connections, it is possible to build a rural community and incorporate individual behavior into the understanding of the rural collective. At the same time, more social network costs make villagers more likely to form local attachments. Building a rural action network and incorporating individual behaviors into public culture for understanding is conducive to promoting residents’ participation in rural environmental governance [37]. Therefore, an increase in the cost of a social network is conducive to rural residents’ choice of moral behavior, rather than completely economic rational behavior.

The objective of a social network refers to the homogeneity and heterogeneity, singleness and diversity, simplicity and complexity of individual communication, reflecting the circle of an individual social network [38]. In a traditional rural society, social networks among residents can be divided into three types: kinship networks, geographical networks and occupational networks, which show strong homogeneity under the limitations of vehicles and modes of transportation [39]. In a closed clan network, the main contacts for residents are family members, neighbors, people in the same clan and clan elders. Under the influence of long-term homogeneous exchanges, the understanding of behavior will be based on the same cultural environment, giving behavior the same value and meaning and forming a habit between villagers, so the behavior of residents will appear to be homogenous, which is conducive to the generation of public behavior [40]. When residents’ social networks expand to the whole village, the value and meaning of individual behavior will be incorporated into the overall village action plan. Residents will include other villagers within the concept of their own men, not others. Therefore, the more social network objectives of villagers tend to be at the rural level, the more they can be encouraged to participate in rural environmental governance.

The frequency of a social network is the number of times an individual participates in formal or informal communication activities within a certain period of time, which reflects the closeness of the connection between the individual and the communication objective [41]. In traditional small-scale farming societies, smallholders are located in certain enclosed spaces. Social network activities are completed in a certain space, with the characteristics of concentration and high frequency. The increase in the frequency of social activities means an increase in the intimacy and depth of communication. The increase in familiarity among members is conducive to establishing a trust mechanism between individuals, obtaining social support from each other and ensuring the high quality of the social network [42]. At the same time, the high frequency of social activities can meet the individual’s emotional needs, help the individual integrate into collective life, place the individual in the emotional society and realize the individual’s socialization, rather than isolating oneself [43]. For rural management, frequent social interaction within the village can enrich the social communication network and structure, create harmonious interpersonal relationships, establish a unique sense of trust in the countryside and reduce the cost of rural environmental governance and mobilization.

The scope of a social network is a geographical concept of communication, which refers to the radius and area of individuals’ social activities [44]. In traditional villages with inconvenient traffic conditions and underdeveloped communication tools, the scope of residents’ social networks is often limited to rural areas. However, under the influence of urbanization, the frequency of communication and interaction between cities and rural areas has increased, and the scope of communication between rural residents has broken the geographical boundaries and realized the interaction between urban and rural areas. The communication of rural residents has moved from the social communication of acquaintances to the social communication of strangers, extending their social communication beyond the countryside and establishing a rich social network. Therefore, after residents move their social contacts out of the village, there is a lack of interaction and communication between them and their previous acquaintances in the village, resulting in the disintegration of the reciprocal relationship. As a result, there is a lack of uniform norms and common interests [45]. At the same time, the concept of urban culture plays a role in infiltrating rural residents. The values and cultural ideas bred by urban culture replace their original rural concepts and cultural traditions. The ideology that tends toward modernity will gradually neglect the original cultural traditions, resulting in ruptures in emotional and cultural identity, and it becomes therefore difficult to achieve a sense of belonging in terms of identity [46]. Therefore, the increase in the range of rural residents’ social networks will not be conducive to their roles in rural environmental governance and will increase the mobilization cost of rural environmental governance.

Based on this, the following hypotheses are proposed:

**Hypothesis** **1** **(H1).** *The cost of a social network has a positive impact on villagers’ participation in environmental governance;*

**Hypothesis** **2** **(H2).** *The objective of a social network has a positive impact on villagers’ participation in environmental governance;*

**Hypothesis** **3** **(H3).** *The frequency of a social network has a positive impact on villagers’ participation in environmental governance;*

**Hypothesis** **4** **(H4).** *The scope of a social network has a negative impact on villagers’ participation in environmental governance*.

### 2.2. Sense of Responsibility

In political science, civic responsibility refers to “a political member’s belief that he or others should participate in the political process without regard to whether the political activity is worthwhile or costly [47]”. A sense of responsibility is the subjective consciousness of the subject regarding the responsibility and is the emotion of the individual to fulfill their own moral obligations actively and seriously [48]. Political responsibility refers to the responsibility and obligation that citizens should assume for national and social development, which is the premise and motivation for citizens to do their own work well [49]. Different from the sense of responsibility in western liberalism, the sense of responsibility in Chinese discourse emphasizes collectivism; that is, the individual’s responsibility to the collective. The sense of responsibility is the intrinsic motivation for residents to take part in rural public activities and collective action. From the perspective of psychology, there exists consistency between psychological consciousness and specific behavior. When individuals have higher responsibility cognition and responsibility emotion, their thoughts and consciousness can be transformed into action and practice. The research results of Kaiser [50] once again confirmed that perception of responsibility, attitude and emotion all affect behavior. Based on this, this paper divides responsibility into four aspects from the perspective of “psychology–behavior” consistency, namely responsibility cognition, responsibility will, responsibility emotion and responsibility behavior.

Responsibility cognition refers to individuals’ cognition of ethics and social responsibility, which is reflected in the division of responsibilities and roles of individuals who place themselves in the rural community and correctly handle the responsibility relationship between individuals and villagers, individuals and rural collectives and individuals and the government [51]. Responsibility will, or responsibility spirit refer to the psychological characteristics formed by individuals who know what rural social responsibility is, consciously participate in the practice of rural public activities, fulfill the responsibility to the collective and transform social responsibility into behavior [52]. Responsibility emotion is a further subjective activity and emotional identity of citizens based on the knowledge of moral responsibility. It is the emotional experience and desire of individuals when they fulfill their responsibility or not [53]. The responsible behavior is the reflection of the subject’s behavior based on the cognition, emotion and will of responsibility [54]. According to the theory of planned behavior, behavioral attitudes, subjective norms, perceived behavioral control and behavioral intentions can all influence individual behaviors [55]. The more intense the rural resident’s consciousness of things they should participate in, the stronger the sense of responsibility, which has a positive effect on the responsible behavior [56]. Based on this, the following hypotheses are proposed:

**Hypothesis** **5** **(H5).** *Responsibility cognition has a positive impact on villagers’ participation in environmental governance;*

**Hypothesis** **6** **(H6).** *Responsibility will have a positive impact on villagers’ participation in environmental governance;*

**Hypothesis** **7** **(H7).** *Responsibility emotion has a positive impact on villagers’ participation in environmental governance;*

**Hypothesis** **8** **(H8).** *Responsible behavior has a positive impact on villagers’ participation in environmental governance.*

**Figure 1:**
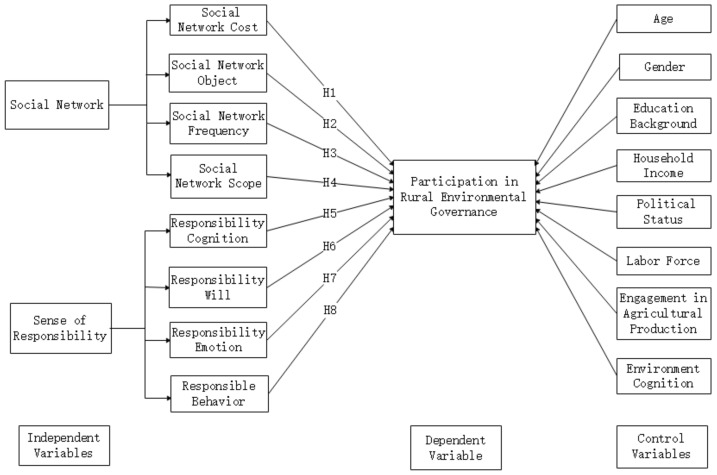
Conceptual model.

Figure 1 presents the conceptual model of hypotheses in this study.

## 3. Methods

### 3.1. Data Sampling

In this study, both questionnaire survey and structured interview were used to analyze the participation in rural environmental governance. The questionnaire mainly includes demographic variables, socio-economic variables, social communication, responsibility and so on. The whole survey follows the principle of random sampling, and the sample size is determined by consultation with experts who are engaged in a large-scale social survey for a long period of time and have rich experience in social surveying. We also draw on the sampling method of some papers using large-scale surveys. There are 34 provincial administrative units in China. Due to the COVID-19 epidemic and the limitation of survey funds, questionnaires were only distributed in 25 provinces in mainland China and were distributed face-to-face by offline researchers. In this study, the sampling survey was adopted to determine the survey objectives. Firstly, 6 cities were selected from one province on average, and one village was chosen from one city, taking into account the economic, regional, population and cultural factors of each province. Secondly, 15 villagers were chosen randomly in each village to conduct a questionnaire survey, with a sample number of 2250. Considering the special circumstances of the Chinese rural area, including the obvious regional economic differences and diversified customs and cultural traditions, some representative villages were included in the survey. 2460 questionnaires were sent and 2343 were received, with the effective 95.24% recovery, which reached the requirement of 90% efficiency. After the questionnaires were collected, 10% of the respondents were randomly selected for a return visit to check the questionnaire data to ensure data authenticity.

Before the questionnaire survey, investigators would explain the survey objective, subject content and purpose to respondents, and the respondent would then finish the questionnaires independently with the consent of the respondent voluntarily and without intervention by the investigators. Each questionnaire took about 15 min, and respondents were compensated with gifts for their help. Meanwhile, the information of all respondents participating in the questionnaire survey remained anonymous and confidential according to the requirements of the survey.

Questionnaire data can be collected in two ways: in one, it is completed independently by the interviewee; in the other, it is completed with the assistance of the researcher. If the interviewee is illiterate, the investigator will complete the survey by semi-structured interview. The investigator explained the purpose and content of the survey to the respondents and asked them if they would participate voluntarily. On the premise of the interviewees’ voluntary participation, the investigators read the questionnaire questions to the interviewees, and the interviewees answered the questionnaire. The investigators should not interfere with the interviewees’ answers or induce them to answer the questionnaire. They should fully respect the interviewees’ answers and record information objectively. It took 15 to 17 min to complete the questionnaire, and participants were also given gifts to compensate for their time and help. To ensure the respondents’ health status, the field research followed the COVID-19 Diagnosis and Treatment Protocol issued by the NHC strictly.

The sample characteristics of 2343 surveyed rural residents are distributed as follows, as shown in Table 2: In terms of regional distribution, the number of respondents in north and south China is 1380 and 963, respectively, accounting for 58.90% and 41.10%. In terms of gender, male respondents accounted for 65.17%; from the perspective of respondents’ age, the respondents under 30 years old, 30–39 years old, 40–49 years old, 50–59 years old,60 years old and above accounted for 4.65%, 7.98%, 19.12%, 30.35% and 37.90%, respectively, and rural residents over 50 years old were the majority. Regarding the occupation of respondents, there are 1523 rural farming residents, accounting for 65%; in terms of marital status, 1976 respondents were married, accounting for 84.34%. In general, the samples reflect the objective situation of rural areas more truly and meet the sampling requirements, which can be statistically analyzed.

### 3.2. Measurements

In this research, rural resident participation behaviors in rural environmental governance have been taken as a dependent variable to investigate the influence of a social network and sense of responsibility on resident participation behaviors in rural environmental governance. Meanwhile, the social network is divided into four dimensions: cost, objective, frequency and scope. Sense of responsibility is divided into four dimensions: cognition, will, emotion and behavior. In addition, factors such as age, gender, education background, household income, political status, labor force, engagement in agricultural production and environment cognition are also used as control variables.

#### 3.2.1. Participation in Rural Environmental Governance

The dependent variable of this paper is rural resident participation in rural environmental governance. The questionnaire is represented as “have you taken part in the cleaning of rural environmental governance”. The answer option has been set as a dichotomous variable, and the sample that answers “no” is assigned 0, and the sample that answers “yes” is assigned 1, as shown in Table 3. Referring to the research results of Hu et al., the participation behavior of rural residents is investigated from a comprehensive perspective. The participation behavior of rural residents in a household garbage treatment, sewage treatment, toilet fecal pollution treatment or village appearance treatment organized by village managers are all considered as the participation behavior of rural residents [57]. According to the actual situation of rural environment governance, there is no fixed mechanism or frequency requirement for rural resident participation, but rural resident participation is visible, while bystanders do not participate. At the same time, this paper examines the voluntary participation behavior of rural residents, and the non-voluntary participation behavior is not included in the scope of investigation because only voluntary behavior has the responsibility factor [58].

#### 3.2.2. Social Networks

A social network is the independent variable in this paper, which is divided into four dimensions (cost, objective, frequency and scope). The first is social network cost. Rural residents’ consumption for maintaining relationships in activities such as marriage, birthdays and festivals is an important reflection of their social network costs. This paper examines the social network costs of rural residents through ritualized interpersonal consumption. According to the Likert five-level scale, the five options have been set for describing consumption expenditure, which are “very low = 1 (200 RMB and below), not too high = 2 (200–500 RMB), general = 3 (500–1000 RMB), relatively high = 4 (1000–2000 RMB), very high = 5 (2000 RMB and above)”. The higher the score, the more the cost of the social network, reflecting that long-term ritual communication constitutes an important cultural practice in villages, which is beneficial to solidarity and mutual support in rural areas [59]. The second aspect is the objective of the social network, which refers to the relationship between residents and their counterparts. The questionnaire was measured from five aspects: parents, neighbors, relatives, friends and villagers. The higher the answer score, the more inclined the social circle is within the rural level. The third is the frequency of social networks, which refers to the network intensity of residents’ interactions [60]. In this paper, the frequency of communication among rural residents is measured, and the five-level Likert scale is also used. The five answers are “never = 1; rarely = 2; general = 3; often = 4; frequent = 5”; the higher the score, the richer the social network. The fourth is the scope of social networks, which refers to the geographical space in which rural residents communicate. Social reach is measured by areas where residents interact frequently. The answers are set according to the regional scope, namely “township = 1; county = 2; city = 3; province = 4; outside the province = 5”. The higher the answer score, the wider the range of social network. Correspondingly, it is easier for residents to gradually break away from the internal rural community.

#### 3.2.3. Sense of Responsibility

Responsibility is another explanatory variable in this paper, which is measured from four dimensions. The first is responsibility cognition. Through the investigation of residents’ cognition of the division of responsibility among the subjects of rural environmental governance—that is, how to share the responsibility among individuals, rural communities and governments [61]—the question is set as to whether they agree to share the responsibility. The answers have been set according to the Likert five-level scale, respectively: “Strongly disagree = 1; Not quite agree = 2; General = 3; Agree = 4; Strongly agree = 5”; the higher the score is, the more sense of responsibility residents have for rural environmental governance. The second is the responsibility will, which is measured by examining whether rural residents are willing to participate in rural environmental governance, and set as a dichotomous variable. The third is responsibility emotion, which is measured by investigating whether rural residents like to participate in public activities and environment governance activities. The option is set as a dichotomous variable, “No = 0; Yes = 1”. The fourth is the responsibility behavior. Whether the rural residents have signed the responsibility letter with the villages for the treatment of rural environmental governance is set as a dichotomous variable, and the value is “No = 0; Yes = 1”.

### 3.3. Analytical Methods

In this study, both descriptive statistics and binary Logistic regression models were adopted for analysis. Firstly, through descriptive statistical analysis, the overall situation of rural resident participation in rural environmental governance was described to understand the general situation of rural resident participation behavior. Secondly, the social network and sense of responsibility of rural residents are described and analyzed to understand the composition of rural residents’ social networks and the distribution of their sense of responsibility. Finally, since the dependent variables in this paper are dichotomous variables, and the independent variables are dichotomous and multi-dichotomous variables, SPSS24.0 (IBM, Armonk, NY, USA) software was employed to explore the influence of a social network and sense of responsibility on villages’ participation activities in environmental governance with the binary logistic regression analysis. The parameters of the model were estimated using maximum likelihood estimation (MLE). The “enter” method was identified as the specific method of operation. Nagelkerke R square and significance were adopted to test the fitting results of the model. Due to the missing values of some variables in the data collection process of the questionnaire, valid samples were used in the specific analysis while the missing values were removed, so the numbers of valid samples used in each step of the analysis show some difference.

## 4. Results

### 4.1. Descriptive Statistics Analysis

Among the 2336 effective samples, 1673 rural residents did not participate in the rural environmental governance, which accounted for 71.62%. The proportion of villagers’ participation in rural environmental governance is less than 30%, and the phenomenon of “bystander rural residents” is serious. The subjectivity and enthusiasm of rural resident participation in rural environmental governance have not been stimulated.

Regarding the social network cost of rural residents, 34.36% of the respondents said that the consumption pressure of ritual favors is relatively high, and another 16.68% of the respondents said that the consumption pressure of ritual favors is very high. The total proportion of the two is more than 50%, which shows that the social interaction structure of acquaintances in traditional villages still exists. The culture, customs and traditions within the village are still the link that maintains the connection between residents and forms a common memory. Ritual communication can provide a certain analytical framework for villagers to understand their own behavior. In terms of social network objectives, 41.16% of the respondents said they had the most contacts with friends, 20.75% said they had more contacts with villagers and 32.71% said they had the most contacts with relatives. In terms of the frequency of social network activities, only 4.95% of the respondents go out frequently, and another 20.11% of the respondents go out a lot. The number of residents going out is not very large, which may be related to the epidemic prevention and control policy in 2021. In terms of the scope of social networks, 30.45% of the respondents indicated that they communicated most in the city, and 11.61% communicated outside the province. This shows that the scope of residents’ contacts has broken the geographical scope of the countryside, moving towards cities or conducting inter-provincial contacts.

Table 4 shows the cross-analysis of social network costs and objectives and villagers’ participation behaviors in rural environmental governance. For villagers whose pressure of interpersonal relation cost were “not too high”, “average”, “relatively high” and “very high”, the corresponding proportions of their participation in rural environmental governance were 23.08%, 23.26%, 29.46% and 40.16%, showing an upward trend. The high consumption pressure of interpersonal relationships indicates that the social network cost of residents is high, which is conducive to building a collective action network in rural areas and increasing social connections. At the same time, among the residents who indicated that their social contacts were “neighbors”, “relatives”, “friends” and “villagers”, the proportions of participating in rural environmental governance were 15.38%, 20.29%, 32.60% and 35.80%, respectively, showing a clear upward trend. It can be found that residents get out of the limitation of family communication and expand their communication circles, especially to communicate with villagers at a wider level, which is conducive to building cultural memory in rural areas and can effectively reduce the cost of rural environmental governance.

In terms of residents’ responsibility cognition, the proportions of “strongly disagree”, “not quite agree”, “general”, “agree”, and “strongly agree” in responding to the division of rural environmental governance responsibilities are 0.17%, 1.54%, 12.26%, 50.09% and 35.94%, respectively, which shows that rural residents have a higher responsibility cognition. From the perspective of responsibility will, 76.09% of respondents are willing to take responsibility for rural environmental governance, while 23.91% are not. For responsibility emotion, only 21% of respondents show their interest in participating in rural public activities, with 79% not interested. The expression of responsible behavior of rural residents is also very low, with only 27.63% of rural residents saying that they had signed the responsibility letter with the rural managers for rural environmental governance, and the remaining 72.37% of respondents did not sign the responsibility letter.

### 4.2. Regression Analysis

Before regression analysis, a multicollinearity test was conducted on all variables with the help of SPSS24.0 software. According to the results, the Durbin–Watson value was 1.834, close to 2, which meant that there existed basically no autocorrelation between those variables. Meanwhile, VIF values of all variables are less than 2, meeting the requirement (ranging from 0 to 10), which indicates that there exists no multicollinearity problem among those variables. In order to clarify the influence of control variables, a social network and sense of responsibility of resident participation in rural environmental governance, the stepwise regression method was used to build the model. In Table 5, control variables are included in the model to generate model 1. Bashed on this, variables of a social network have been included to build model 2, and then responsibility variables have been incorporated to build model 3. All of these models’ results were significant (Sig = 0.000), and Nagelkerke R square rose from 0.149 to 0.314, showing that the model fitting effect is gradually improving, and that the fitting validity is acceptable for social sciences [62]. From model 1 to model 3, the −2 log-likelihood value shows a downward trend, decreasing by 390.043 and indicating that the model fitting effect is improving. The results of the Hosmer–Lemeshow test are all greater than 0.05, and the test result of model 3 is 0.755, indicating that the model fitting results are acceptable.

The level of education has always influenced resident participation in rural environmental governance. In Model 3, when residents’ education improves by one unit, the probability of them taking part in environmental governance increases by 1.175 times. Household income significantly affects rural resident participation in environmental governance. In model 3, when household income level increases by one unit, the probability of villagers taking part in environmental governance will be 0.825 times than not taking part. Residents with higher household income are more rational and more likely to choose not to participate, which is the same as existing research. Political status has a significant effect in Model 1, but has no significant effect in Model 2 and Model 3, indicating that its effectiveness is unstable. The labor force factor has a significant effect. In Model 3, each additional unit of labor force increases the probability of villagers to take part in rural environmental governance by 1.191 times. More household labor provides more possibilities to participate in environmental governance. Engagement in agricultural production has a major influence. Residents engaged in agricultural production are 1.717 times more likely to take part in rural environmental governance than residents who did not engage in agricultural production. Environmental cognition has a significant influence on villagers’ participation activities in environmental governance. With each additional unit of environmental cognition, the probability of villagers taking part in rural environmental governance will rise by 1.484 times.

In the third model, variables included in a social network and sense of responsibility greatly influence resident participation in rural environmental governance. The effect of each variable is arranged in descending order as follows: responsibility emotion, responsibility behavior, responsibility will, social network cost, responsibility cognition, social network frequency, social network objective and social network scope. The specific analysis is as follows: There exists a significant positive impact between villagers’ responsible emotion and their participating behaviors in rural environmental governance, and the impact effect is 1.122. Compared with residents who do not like to take part in public activities, the residents who like to participate in rural public activities are 3.07 times more likely to take part in environmental governance. The influence of responsibility behavior on resident participation in rural environmental governance is 1.037. Compared to the residents who did not sign the responsibility letter for rural environmental governance, the residents who signed the responsibility letter were 2.821 times more likely to participate in the rural environmental governance. There is a significant positive impact of responsibility will on resident participation in rural environmental governance, with an impact effect of 0.411. Compared with residents who are unwilling to participate in rural environmental governance, the probability of willing residents to participate in rural environmental governance is 1.509 times higher.

Social network cost has a positive and significant influence on villagers’ participation in rural environmental governance, with an impact coefficient of 0.264. With each unit increased in the social network cost of residents, the probability of participating behaviors in environmental governance will rise by 1.302 times. Residents’ spending on social networks can help to form a rural social network system, increase information sharing among residents, obtain relevant policy information on rural environmental governance and play a leading role in the demonstration of acquaintances. Inter-village exchanges increase rural cultural identity and interest linkages, which is an important basis for villagers to participate in social activities. Responsibility cognition has a positive and significant influence on rural resident participating behaviors in environmental governance, with an impact coefficient of 0.250. When residents’ awareness of responsibility increases by one unit, the probability of their participation in environmental governance will rise by 1.284 times. The frequency of a social network has a positive and significant influence with an influence coefficient of 0.169. With each unit increased in the frequency of residents’ interaction, the probability of their participation increases by 1.185 times. However, social network scope has a negative influence with an impact coefficient of −0.143. With each additional unit in rural residents’ communication scope, their probability to participate is 0.866 times that of not participating. The long-term trans-regional communication of rural residents will make them gradually break away from the original rural community, reducing both their attachment to the countryside and their concern for rural public affairs. Social network objectives have a positive and significant influence with an influence coefficient of 0.136. The probability of participation increases by 1.145 times with each additional unit of resident’s contacts.

Finally, the results of hypotheses testing are shown in Table 6, H1, H2, H3, H4, H5, H6, H7 and H8 and have all been verified.

## 5. Discussion

In order to study the influence of a social network and sense of responsibility on residents’ participation in rural environmental governance, this paper adopted the binary logistic regression model as an analysis tool to explore the relationship among the three elements. The stepwise regression model results show that both social networks and sense of responsibility significantly influence resident participation in rural environmental governance, which proves the hypotheses H1, H2, H3, H4, H5, H6, H7 and H8 proposed in this paper. The social network activities in which rural residents participate, especially within rural areas, can strengthen the relationship and interests of residents. The cultural identity and emotional identity generated by social network activities provide a valuable framework for understanding individual behavior [63]. The individual behavior understood in the interaction is endowed with collective value and meaning. However, when residents’ social activities go beyond rural boundaries, their sense of identity with the village will be reduced. As a result, they are less concerned about public activities in rural areas. Residents’ sense of responsibility reflects their cognition of the rights and obligations of public affairs in rural areas and becomes the internal driving force for them to participate in environmental governance.

Hypothesis 1 has been confirmed where the cost of social network activities has a positive and significant influence on rural residents’ participation behavior. This is different from the findings of Qiu [64] et al., who argue that an increase in the cost of a social network reduces further social interaction and hinders the generation of governance behaviors. This paper argues that the increase in the cost of social network reflects the frequent interaction among rural residents, which is conducive to the formation of social network structure. Ritual environments place individuals in communication and interaction and have an interactive social nature. This ritual carrier not only provides conditions for individuals to obtain information and resources, but also increases the degree of intimacy between individuals. It is characterized by reciprocity, giving social meaning to individual actions [65]. In view of social interaction theory, the repetitiveness of internal interactions can share a cultural idea or pattern with others. This pattern creates an emotional bridge between interactors, which in turn prompts the interactors to produce the same social behavior [66]. This research has a certain universality for the entire Asian region. Under the influence of Confucian humanism, the reciprocal effect of a social network is emphasized, and the establishment of “warm” interpersonal relations promotes individuals to participate in collective actions [67].

Research Hypothesis 2 has been proved in that residents’ social network objectives have a positive and significant impact on their participation behavior, which is consistent with the research results of Nygren [68]. However, the result is different from Olli’s research. He believes that the expansion of communication objectives will form weak ties which are not conducive to the production of friendly environmental behaviors [69]. It is easy for rural residents to form a strong relationship network with their family members and neighbors, while a weak relationship network is formed with other villagers which reflects the phenomenon that their communication objectives present a differential pattern from near to far [70]. When the social network objectives of rural residents expand from families to higher-level villagers, they establish network connections with other villagers to realize information exchange, resource exchange and emotional transmission with other villagers. As a result, their focus on things will gradually expand from the family to the countryside, and their awareness of the community of interests and life will increase [71]. Individuals integrating the development of self and family at the rural level, rather than the development of a single subject, can get rid of the vulnerability of small household farmers for collective behavior in rural areas, participation in rural collective action is the premise of overcoming isolation and vulnerability.

Research Hypothesis 3 has proved that the frequency of social network activities of villagers has a positive and significant impact on their participation behavior, which again supports the findings of Plummer et al. [72]. Long-term communication and interaction between villagers can establish stable relationships, making it possible for a mutually beneficial relationship and trust mechanism to persist between them. From an ideological point of view, long-term superimposed exchanges can generate trust in others in personal ideology and expand the trust of relatives and neighbors to the trust of villagers and villages, increasing the overall trust of the entire village [73]. The trust between villagers can endogenously generate a certain emotional identity and custom identity, generate local attachment to the countryside and then promote individual participation in rural public activities [74]. Residents are important participants and subjects of rural environmental governance. The interactive structure brought about by frequent social interaction can maintain the enthusiasm and positive attitude of farmers to participate in rural public affairs, which is conducive to the formation of a sustainable environmental governance system [75]. For village managers, trust among villagers can reduce the cost of governance and improve the efficiency of environmental governance.

Research Hypothesis 4 is confirmed, according to which residents’ social network scope has a significant negative influence on villagers’ participation behavior. Thomas Macias holds that the closeness of the relationship has an impact on the attitude of residents to participate in rural environmental governance [76]. On this basis, this study further points out that the weak relationship network formed after the expansion of residents’ social interaction is not conducive to their participation in rural environmental governance. When the countryside is involved in the process of urbanization, rural residents will gradually leave the countryside and migrate to the city, which means that they begin to leave the rural community. The connection between the residents who enter the city, and their original rural social interaction will be reduced, and the weak relationship with the rural members will be formed. As a result, it is easier for them to place their behavior in the external environment to understand and to give it the meaning of modern urban culture [77]. Their cultural identity concept will also be affected. By learning modern urban culture, they will gradually drop the backward rural culture and maintain differences in value concept and cultural identity [78]. Therefore, the scope of social network activities has an inhibitory effect on residents’ participation in rural environmental governance.

Hypothesis 5 confirms that rural residents’ perception of responsibility has a significant positive impact on participation. This is consistent with the research results of Shen et al. [79]. The main body of rural public environmental governance consists of rural governments, rural managers and rural residents who play different roles and take on respective responsibilities. Only when the residents who account for the majority of the rural population correctly recognize the environmental governance responsibility they should undertake—and also internalize the rural collective responsibility into individual responsibility—can they be stimulated by a higher responsibility cognition to participate in public activities. The essence of residents’ responsibility cognition is to recognize the significance and value of environmental governance behavior, enhance their self-efficacy and stimulate their initiative. The government’s environmental governance publicity can also enhance rural residents’ awareness of responsibility, sense of responsibility and mission and encourage individuals to take the initiative to take responsibility and fulfill their environmental governance responsibilities [80].

Hypothesis 6 confirms that rural residents’ will in undertaking responsibility has a significant positive impact on their participation behavior. This is different from the findings of Wallington et al. [81]. From the perspective of “attitude-behavior” consistency, unless constrained by external coercive forces or restrictive conditions, residents with a strong will to responsibility have a very small probability of taking on non-participation behaviors [82]. This study has found that residents who were not coerced had inconsistent wishes and behaviors. Residents have a strong will to take responsibility, but their actual participation in rural environmental governance is very low. In essence, the will to responsibility is the ability of an individual to recognize and control his own behavior, and it expresses the individual’s subjective will to produce behavior. The possible explanations are as follows: residents have limited time to participate, or are busy with agricultural production [83], or the ordering of demands at the same time makes environmental governance unimportant [84], or village managers do not get properly organized. From the sustainable perspective of environmental governance, residents with a high will to take responsibility can promote the sustainable development of environmental governance [85].

Hypothesis 7 confirms that rural residents’ emotion of responsibility has a significant positive impact on their participation behavior. This once again proves the research results of Dumreicher et al. [86]. In essence, the responsibility emotion is the residents’ identification with the rural culture and the rural community, which closely connects individuals, families and rural development. Individuals who actively care about rural public affairs are willing to make individual contributions to rural environmental governance and produce responsible altruistic behavior [87]. Residents who have local attachment plots in their rural life have certain feelings and a sense of gain for the countryside and will naturally develop feelings of individual responsibility. Responsibility emotion requires individuals to break the limitations of family and neighbors, expand their sense of responsibility to the rural level and fulfill their responsibilities for rural development. The level of responsibility emotion indicates the individual’s love for rural life. Placing the development of the countryside above the interests of individuals is an intrinsic sublimation of the cognition of responsibility, and it is also the value and significance of enhancing individual responsibility [88].

Hypothesis 8 confirms that rural residents’ responsibility behavior has a significant positive impact on their participation behavior. The system implemented by village managers can stimulate rural resident participation, which is similar to the findings of Deininger et al. [89]. According to Habermas’ social interaction theory, individual behavior is influenced by institutions. For rural managers, the establishment of a certain environmental governance system helps to restrict individual behaviors. Through signing treaties with residents to fulfill their responsibilities, contractual relationships are formed, and rural residents’ behaviors are constrained, which will greatly reduce the cost of environmental governance [90]. The important role of residents’ signing behavior is to restrict their behavior. They need to be accountable to the contracted system, thereby reducing non-participation [91]. However, their signing must be a voluntary choice, not a forced one. From the perspective of planned behavior theory, the path of responsible behavior is “subjective norm- behavioral intention- behavior”. Therefore, behavior is the result of the development of responsibility cognition, responsibility will and responsibility emotion [92].

## 6. Conclusions

Based on a survey of 2343 Chinese rural residents, this paper adopted the binary logistic regression model to explore the impact of a social network and sense of responsibility on resident participation in rural environmental governance. The research shows that both social networks and a sense of responsibility have a significant impact on resident participation.

Hypotheses H1, H2, H3, H4, H5, H6, H7 and H8 have been confirmed. Social network costs reflect residents’ efforts to build network relationships and increase mutual understanding through ritualized interpersonal activities. Social network objectives reflect the circle of interpersonal communication. Transforming weak ties at the rural level into strong ties, changing residents’ consideration of individual and family interests and improving the status of rural public interests will help to promote the emergence of rural public behavior. An increase in the frequency of social network activities is conducive to the establishment of a relational society, increasing the trust between members and promoting the emergence of a community based on geography and blood ties. Social network activities are conducive to the organization of public affairs by building the collective memory of rural areas and integrating factors such as culture, customs and emotions into the values of rural residents. However, social interactions beyond rural areas will disintegrate the traditional acquaintance society, which is not constructive to villagers’ formation of rural cultural identity, custom identity and emotional identity. As a result, rural communities will disintegrate, reducing both rural residents’ attention to rural affairs and their participation behavior. As the intrinsic driving force of rural resident participation, the sense of responsibility plays an important role and constitutes the intrinsic driving force of rural resident participation. The sense of responsibility also makes rural environmental governance resilient. However, there is an inconsistency between residents’ responsibility will and responsibility behavior which makes it difficult to generate responsible altruistic behaviors in practice. Therefore, this disadvantage must be corrected to promote the sustainable development of rural environmental governance.

Based on research results, the following suggestions are put forward: Firstly, social network activities in rural areas should be enriched. Rural managers can make full use of ritual communication activities, social organization activities and holiday activities to carry out social network activities in various forms and with rich content in order to increase the emotional connection among rural residents. Secondly, we should also make full use of internet technology to strengthen the connection with rural residents who have moved out of the rural areas. We should extensively and deeply publicize the progress of rural environmental governance through social network applications including Wechat, Tiktok, QQ and other online means, increase the cultural connection and interest connection between rural residents and rural areas, attracting their participation. Thirdly, the role of the villagers meeting and assembling should be brought fully into play to collect opinions on the governance of rural environment from rural residents to play their role as masters and enhance their sense of responsibility and participation. Finally, it is also necessary to establish a rural environmental protection publicity group to regularly publicize environmental protection policies to residents, so as to enhance the environmental protection awareness and sense of responsibility of rural residents and to stimulate their endogenous motivation.

There were four limitations in this study: Firstly, the data of this research is only from one survey, and there is not enough continuous tracking data, so the data in this paper only reflects the present situation. Secondly, rural resident participation behavior is the result of comprehensive factors, so the influence of other variables on rural resident participation behavior also needs to be investigated to improve the fitting degree of the model. Thirdly, there is much content in rural environmental governance, but this paper is only a comprehensive investigation of rural resident participation behavior and lacks an investigation of specific aspects of each behavior, as well as the willingness to participate in specific aspects. Finally, the relationship between sense of responsibility and the social network has not been explored in depth, which results in less contribution to the study of a sense of responsibility. It also remains to be proved whether social networks within rural areas will improve rural residents’ sense of responsibility. Since China is a country with vast territory, differences in participation among provinces are also worth discussing.

## Figures and Tables

**Table 1:** Rural economic development (Unit: 100 million RMB).

Index	Year				
	2016	2017	2018	2019	2020
Gross output value of agriculture, forestry, animal husbandry and fishery	106,478.73	109,331.72	113,579.53	123,967.90	137,782.17
Gross agricultural output value	55,659.89	58,059.76	61,452.60	66,066.50	71,748.23
Gross forestry output value	4635.90	4980.55	5432.61	5775.70	5961.58
Gross animal husbandry output value	30,461.17	29,361.19	28,697.40	33,064.30	40,266.67
Gross fishery output value	10,892.92	11,577.09	12,131.51	12,572.40	12,775.86
Per capita disposable income of rural residents (RMB)	12,363	13,432	14,617	16,021	17,131
Per capita disposable wage income (RMB)	5022	5498	5996	6583	6974

Data source: Official website of China National Bureau of Statistics.

**Table 2:** Characteristics of the survey sample.

Characteristic Index	Classification	Frequency	Proportion (%)	Standard Deviation
Area	North	1380	58.90	0.49
	South	963	41.10	
Gender	Male	1527	65.17	0.48
	Female	816	34.83	
Age	Under the age of 30	109	4.65	1.14
	30–39	187	7.98	
	40–49	448	19.12	
	50–59	711	30.35	
	Aged 60 and above	888	37.90	
Occupation	Agriculturalists	1523	65.00	1.60
	Migrant workers	372	15.88	
	Rural teachers	29	1.24	
	Self-employed and private business owners	154	6.57	
	Rural administrators	58	2.48	
	Else	207	8.83	
Marital status	Single	134	5.72	0.62
	Married	1976	84.34	
	Divorced	41	1.75	
	Widowed	192	8.19	
In total		2343	100	

**Table 3:** Variable definition and assignment.

Variable Types	The Variable Name	Variable Definitions	Mean Value	Standard Deviation
Dependent variable	Participating in rural environmental governance	No = 0; Yes = 1	0.28	0.45
Control variable	Age	Under 30 = 1; 30–39 = 2; 40–49 = 3; 50–59 = 4; 60 and over = 5	3.89	1.14
	Gender	Female = 0; Male = 1	0.65	0.48
	Education background	Illiteracy = 1; Primary school = 2; Junior school = 3; High school = 4; college or above = 5	2.65	0.95
	Household income	Low income = 1; Low and middle income = 2; Middle income = 3; Upper middle income = 4; High income = 5	2.94	1.39
	Political status	Non-party member = 0; Party member = 1	0.09	0.286
	Labor force	The number of labor force of the interviewed family, the value range is 0–7	2.33	1.234
	Engagement in agricultural production	No = 0; Yes = 1	0.64	0.481
	Environment cognition	Little knowledge = 1; Some knowledge = 2; Sufficient knowledge = 3	1.83	0.852
Social network	Social network cost	Very low = 1; Not too high = 2; General = 3; Relatively high = 4; Very high = 5	3.56	0.93
	Social network objective	Parents = 1; Neighbors = 2; Relatives = 3; Friends = 4; Villagers = 5	3.76	0.86
	Social network frequency	Never = 1; Rarely = 2; General = 3; Often = 4; Frequent = 5	2.82	0.98
	Social network scope	Township = 1; County = 2; City = 3; Province = 4; Outside the province = 5	3.73	0.724
Sense of responsibility	Responsibility cognition	Strongly disagree = 1; Not quite agree = 2; General = 3; Agree = 4; Strongly agree = 5	4.20	0.72
	Responsibility will	Unwilling = 0; Willing = 1	0.76	0.43
	Responsibility emotion	No = 0; Yes = 1	0.28	0.45
	Responsibility behavior	No = 0; Yes = 1	0.28	0.45

**Table 4:** Cross analysis of social network and resident participation in rural environmental governance (Units: %).

Social Network Cost	Participating in Rural Environmental Governance		Social Network Objective	Participating in Rural Environmental Governance	
	No	Yes		No	Yes
Very low	67.27	32.73	Parents	80.00	20.00
Not too high	76.92	23.08	Neighbors	84.62	15.38
Average	76.74	23.26	Relatives	79.71	20.29
Relatively high	70.54	29.46	Friends	67.40	32.60
Very high	59.84	40.16	Villagers	64.20	35.80
Sample: 2308; *p* = 0.000			Sample: 2336; *p* = 0.000		

Note: *p* is the result of Pearson’s chi-square test.

**Table 5:** Regression analysis of a social network, sense of responsibility and resident participation in rural environmental governance.

Variate	Model 1		Model 2		Model 3	
	β	Sem	β	Sem	β	Sem
Age	−0.050	0.049	−0.058	0.051	−0.054	0.054
Gender (female)	0.115	0.107	0.147	0.111	0.113	0.118
Education background	0.147 *	0.061	0.185 **	0.063	0.162 *	0.067
Household income	−0.098 *	0.039	−0.122 **	0.041	−0.192 ***	0.045
Political status (non-party member)	0.445 **	0.166	0.312	0.173	0.231	0.193
Labor force	0.158 ***	0.044	0.161 **	0.047	0.174 ***	0.051
Engagement in agricultural production (no)	0.555 ***	0.109	0.558 ***	0.113	0.540 ***	0.121
Environment cognition	0.697 ***	0.058	0.629 ***	0.060	0.395 ***	0.065
Social network cost			0.252 ***	0.055	0.264 ***	0.060
Social network objective			0.282 ***	0.062	0.136 *	0.068
Social network frequency			0.364 ***	0.076	0.169 **	0.082
Social network scope			−0.171 **	0.054	−0.143 *	0.058
Responsibility cognition					0.250 **	0.085
Responsibility will (unwilling)					0.411 **	0.149
Responsibility emotion (no)					1.122 ***	0.131
Responsibility behavior (no)					1.037 ***	0.117
Constant	−3.059 ***	0.354	−5.831 ***	0.539	−8.519 ***	0.659
Model fit	0.000		0.000		0.000	
−2 log-likelihood	2520.745		2387.136		2130.702	
Nagelkerke R squared	0.149		0.201		0.314	
Hosmer-Lemeshow test	0.054		0.110		0.755	
Valid sample	2326		2290		2254	

Note: 1. * *p* ≤ 0.05, ** *p* ≤ 0.01, *** *p* ≤ 0.001; 2. reference group in parentheses; 3. The values in the table are coefficients β, OR = exp(β).

**Table 6:** Results of hypotheses testing.

Hypotheses	Results
H1: Social network cost → Resident participation in rural environmental governance (positive)	Cannot be rejected
H2: Social network objective → Resident participation in rural environmental governance (positive)	Cannot be rejected
H3: Social network frequency → Resident participation in rural environmental governance (positive)	Cannot be rejected
H4: Social network scope → Resident participation in rural environmental governance (negative)	Cannot be rejected
H5: Responsibility cognition → Resident participation in rural environmental governance (positive)	Cannot be rejected
H6: Responsibility will → Resident participation in rural environmental governance (positive)	Cannot be rejected
H7: Responsibility emotion → Resident participation in rural environmental governance (positive)	Cannot be rejected
H8: Responsible behavior → Resident participation in rural environmental governance (positive)	Cannot be rejected

## Data Availability

The data presented in this study are available on request from the author (ruanhb@mails.ccnu.edu.cn). The data are not publicly available due to privacy reasons.
